# Safety, tolerability and immunogenicity of V934/V935 hTERT vaccination in cancer patients with selected solid tumors: a phase I study

**DOI:** 10.1186/s12967-020-02228-9

**Published:** 2020-01-30

**Authors:** Luigi Aurisicchio, Arthur Fridman, David Mauro, Rose Sheloditna, Alberto Chiappori, Ansuman Bagchi, Gennaro Ciliberto

**Affiliations:** 1 Takis Biotech, Rome, Italy; 2 Merck & Co., Inc., Kenilworth, NJ USA; 3 Prelude Therapeutics, Wilmington, DE USA; 4 Celgene Corporation, Summit, NJ USA; 5 Department of Thoracic Oncology, Moffitt Cancer Center and Research Institute, Tampa, FL USA; 6 Scientific Directorate, Regina Elena National Cancer Institute, IRCCS, Rome, Italy

**Keywords:** hTERT, Cancer vaccination, Adenovirus, Electroporation, Prime-boost immunization

## Abstract

**Background:**

Human telomerase reverse transcriptase (hTERT) is an antigen that may represent a target for a novel anti-cancer strategy. A pilot, phase I study tested the safety and feasibility of a prime-boost immunization regimen based on V935, an adenoviral type 6 vector vaccine expressing a modified version of hTERT, administered alone or in combination with V934, a DNA plasmid that also expresses the same antigen and is delivered using the electroporation injection technique.

**Methods:**

Treatments: Group #1 received two doses (low-dose: 0.5 × 10^9^ vg, and high-dose: 0.5 × 10^11^ vg) of V935 followed by a 4-week observation period. Group #2 received three doses of V934-electroporation and two doses of V935 following a 4-week observation period. Doses were low-dose V934 (0.25 mg of plasmid) with low-dose V935 (0.5 × 10^9^ vg); high-dose V934 (2.5 mg of plasmid) with high-dose V935 (0.5 × 10^11^ vg). Group #3 received five doses of V934-EP and two doses of V935: V934 was administered IM every 2 weeks for five doses. Following a 4-week observation period, V935 was administered IM every 2 weeks for two doses followed by a 4-week observation period. One (1) dose level was tested in treatment group #3: high-dose V934 (2.5 mg of plasmid), in combination with high-dose V935 (0.5 × 1011 vg). Immunogenicity was measured by ELISPOT assay and three pools of peptides encompassing the sequence of hTERT.

**Results:**

In total, 37 patients affected by solid tumors (prostate cancer in 38%) were enrolled. The safety profile of different regimens was good and comparable across groups, with no severe adverse events, dose-limiting toxicities or treatment discontinuations. As expected, the most common adverse events were local reactions. A significant increase in ELISPOT responses against hTERT peptide pool 2 was observed (p < 0.01), while no evidence of boosting was observed for peptide pools 1 and 3. This was also evident for group #1 and #2 separately. In patients with prostate cancer, there was a significant increase in ELISPOT response against hTERT peptide pool 2 following immunization (p < 0.01), regardless of previous therapy, immunosuppressing agents, or adenoviral type 6 titers at screening.

**Conclusion:**

Our results suggest the safety and feasibility of V934/V935 hTERT vaccination in cancer patients with solid tumors

*Trial Registration* Name of the registry: ClinicalTrial.gov Trial registration number: NCT00753415 Date of registration: 16 September 2008 Retrospectively registered URL of trial registry record: https://clinicaltrials.gov/ct2/results?cond=&term=NCT00753415&cntry=&state=&city=&dist=

## Background

Cancer vaccination has been regarded as a safe and potentially promising anti-tumor strategy [1–6]. Nonetheless, the vast majority of peptide/protein and cell-based vaccines failed to induce clinically relevant immune responses in patients who present a more severe disease [7]. Tumor vaccines based on local gene delivery (genetic vaccines) using attenuated recombinant viral vectors (including adenovirus) or naked DNA plasmid-based vectors have been developed to improve the immunogenicity and clinical efficacy of existing peptide or cell-based approaches [8–10]. Adenoviral vectors have shown to be quite efficient in generating cell-mediated immunity against foreign antigens based on pre-clinical and clinical studies; however, a major feature of adenoviral vectors is their capacity to rapidly induce a neutralizing antibody response to capsid proteins, which strongly limits the use of repeated vaccinations [11]. On the other side, development of DNA plasmid-based cancer vaccines has been hampered by the poor effectiveness of gene transfer [7]. Pleasingly, combining the injection of a plasmid vaccine with electroporation (EP) increases the efficiency of gene expression and the induction of immune responses in pre-clinical studies compared with DNA vaccination alone, thus potentially circumventing the DNA degradation issue [12, 13]. Trials with adenoviral vectors in clinical gene therapy showed a tumor response with minimal safety issues [14–16]. Furthermore, “heterologous prime-boost” immunization regimens that combine sequential vaccination with two different gene-based delivery systems (plasmid DNA and adenoviral vectors) have been developed, and this approach can generate a more intense and durable immune response [12].

Telomerase is a ribonucleoprotein reverse transcriptase complex (hTERT) containing an RNA subunit and a protein catalytic subunit that is involved in maintaining telomeric DNA and thereby inhibiting replicative senescence [17, 18]. hTERT expression and telomerase enzymatic activity are absent in most human adult somatic cells. In contrast, increased telomerase enzymatic activity and/or hTERT expression have been noted in a wide range of malignant cells, suggesting that hTERT could serve as an attractive target for vaccine-based approaches [19, 20]. Notably, hTERT can elicit cytotoxic lymphocytes that lyse tumors of various histologic types [21, 22]. hTERT is, therefore, a frequent tumor-associated antigen and efforts at enhancing immune reactivity against hTERT may hold substantial promise as a novel anti-cancer strategy.

This pilot, phase I study tested the safety and feasibility of a novel prime-boost immunization regimen consisting of two doses of V935, an adenoviral type 6 vector [Ad6] vaccine expressing hTERT, administered alone or in combination with three to five doses of V934, a DNA plasmid that also expresses hTERT and is delivered using a novel in vivo EP injection technique.

## Methods

### Vaccine

V935 is an Adenovirus-serotype 6 vector encoding a catalytically inactive version of hTERT [23]. V934 is a DNA plasmid vaccine encoding a codon optimized, catalytically inactive hTERT protein fused to a TPA (human tissue plasminogen activator) leader sequence at N-term and fused itself to the β-subunit of *Escherichia coli* heat labile enterotoxin (LTB) at C-term. For both vectors, transcription is controlled by the human cytomegalovirus (CMV) major immediate early (IE) enhancer/promoter and is terminated by the bovine growth hormone (bGH) polyadenylation signal. The underlying study for this work was registered as NCT00753415 at the ClinicalTrials.gov web site.

### Study design and vaccine schedule

This was a phase I, multicenter, open-label trial (NCT00753415) in patients with selected solid tumors. The study was conducted in accordance with principles of the Declaration of Helsinki, in compliance with Good Clinical Practice (GCP), and approved by the appropriate institutional review boards and regulatory agencies.

**Fig. 1:**
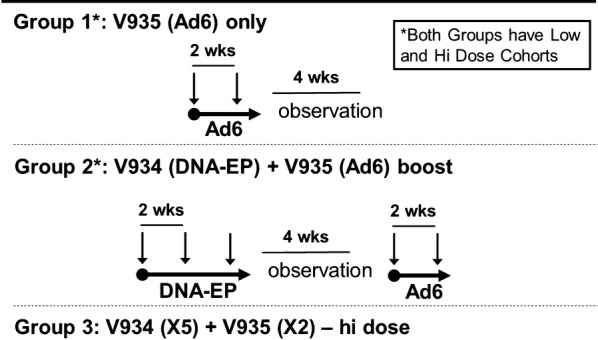
Study design

The study consisted of two parts (see Fig. 1). In part A, patients were assigned in an alternating fashion to one of two treatment groups, each consisting of a low- and a high-dose cohort. In part B, an optional boost was offered to all part A participants, once they completed all vaccinations in their respective treatment groups. However, we present only the results of part A in this manuscript, for the sake of clarity.

The groups analyzed in part A were as follows: Group #1, V935 alone: administered intramuscularly (IM) every 2 weeks for two doses followed by a 4-week observation period. Dose level cohorts were: low-dose V935 (0.5 × 10^9^ vg) and high-dose V935 (0.5 × 10^11^ vg). Group #2: V934-EP and V935: V934 was administered IM every 2 weeks for three doses. Following a 4-week observation period, V935 was administered IM every 2 weeks for two doses followed by a 4-week observation period. Dose level cohorts were as follows: low-dose V934 (0.25 mg of plasmid) in combination with low-dose V935 (0.5 × 10^9^ vg); then high-dose V934 (2.5 mg of plasmid), in combination with high-dose V935 (0.5 × 10^11^ vg). Within each group, patients were enrolled first in a low-dose group and then, in a high-dose group using a 3 + 3 design. High-dose cohorts were then expanded to further assess the safety, tolerability and immunogenicity of the regimen. Each treatment group was evaluated independently. A third treatment group (group #3) was also evaluated, following a review of the safety and immunogenicity data from the initial two treatment groups. Group #3 received V934-EP and V935: V934 was administered IM every 2 weeks for five doses. Following a 4-week observation period, V935 was administered IM every 2 weeks for two doses followed by a 4-week observation period. Doses were high-dose V934 (2.5 mg of plasmid), in combination with high-dose V935 (0.5 × 10^11^ vg).

V934 was administered as a 0.5 mL injection given intramuscularly, at a 90° angle, into the deltoid muscle of alternating arms using a 1.0 mL syringe with a 27-gauge, 1.27-cm needle. Within 2 min of each injection of V930, each patient was given an EP IM injection consisting of two 60 ms pulses using the MedPulser™ DDS device.

#### Dose-limiting toxicity and maximum tolerated dose

Adverse events (AEs) were graded according to the CTCAE, version 3.0 guidelines and dose-limiting toxicity (DLT) were defined as vaccine- or EP-related AEs as follows: grade 4 neutropenia that lasts > 5 days; grade 3 neutropenia with fever > 38.5 °C; grade 4 thrombocytopenia (> 25 × 10^9^/L); any grade 3 or 4 non-hematologic toxicity, except alopecia, inadequately treated grade 3 nausea, vomiting or diarrhea that lasts less than 48 h, grade 3–4 or 4 CPK elevations, not associated with rhabdomyolysis or isolated grade 3 or 4 CPK elevations that last more than 6 days; inadequately treated hypersensitivity reactions; uncomplicated grade 3 elevations of liver enzymes lasting less than 1 week.

The maximum tolerated dose of V934/V935 was defined as the dose level immediately below that in which two patients of a group experienced a DLT.

#### Dose-escalation scheme

If no DLTs was seen in the first three low-dose patients, for a given treatment group, dose escalation was performed, with six new patients enrolled in the high-dose cohort of the same treatment group. If one DLT was detected, three more patients were enrolled in the same low-dose group, for a total of six patients. If two or more DLTs were seen in the six low-dose patients, enrollment in that treatment group was stopped. If only one DLT was seen in the expanded six low-dose patients, dose escalation continued with up to six new patients enrolled in the high-dose cohort of that treatment group.

In the high-dose groups, if 0 or one DLT out of the initial six patients were seen, four more patients for a total of ten patients were enrolled, to further evaluate safety, tolerability, and immunogenicity of the regimen. If two or more DLTs were seen, the high dose was not considered tolerable, and a cohort expansion to a total of ten patients occurred at the low dose for that treatment group.

### Study population

A written informed consent was obtained from all patients prior to study participation. Adult (≥ 18 years) patients of either gender with diagnosis of different types of solid tumors, ECOG performance status of 0/1; adequate bone marrow, hepatic and renal functions; negative serum pregnancy test within 3 days of study enrollment for women of childbearing potential.

Patients were excluded if they met the following criteria: previous therapy with any hTERT-containing/targeted vaccine or any adenoviral or DNA vaccine; HIV infection or hepatitis B or C; any active medical condition (e.g., arrhythmia or myocardial infarction) within the last 3 months; ICD and/or pacemaker; active psychiatric or substance abuse disorder; history of splenectomy or autoimmune disorders; immunosuppressive therapy; history of coagulopathy or thrombocytopenia prohibiting IM injections; symptomatic ascites or pleural effusion; allergy to any of the vaccine components or history of a second malignancy.

### Study procedures

Patients recorded AEs on an Adult Treatment Report Cards (ATRC) daily for 14 days following any vaccination. Patients were followed for at least 30 days following their last dose of study therapy. Patients were monitored for the development of AEs and were monitored for evidence of disease progression according to the institutional standards of clinical practice using good clinical judgment and when appropriate, the Response Evaluation Criteria In Solid Tumors (RECIST) criteria.

### Laboratory assays

Immunogenicity of the vaccine was primarily measured using the ELISPOT (enzyme-linked immunospot) assay. 3 pools of 15-mer peptides (pool 1, 2 and 3), overlapping by 11 residues and encompassing the entire hTERT were utilized. A pool covering the LTB sequence was used as well. Measurements were performed at baseline (screening and pre-vaccination day 1), and various time points post-vaccination (week 3; week 5; week 7; week 10; week 12; week 14; week 18; week 20; week 24; and week 30). A positive ELISPOT response was defined by a minimum number of spot-forming cells per million peripheral blood mononuclear cells (SFC/10^6^ peripheral blood mononuclear cells) for the antigen well and a minimum n-fold increase in the antigen well with respect to the control well.

### Statistical analysis

Based on the trial design, the maximum number of evaluable patients needed in this study is (6 + 10) × 2 = 32. Since the optional third treatment group also enrolled patients, then the maximum number of evaluable patients to be enrolled became 32 + 10 = 42 (only the high-dose level of the third treatment group was investigated).

Data were analyzed by descriptive statistics. Inter- and intra-group comparisons were performed by the paired *t* test or Wilcoxon signed-rank test, as appropriate. Data were analyzed in the overall population, by treatment group and by tumor subtype. A p-value < 0.05 was considered statistically significant.

## Results

### Patient characteristics

In total, 37 patients were enrolled (mean age 62 ± 11 years; range 36–83; 27 males, 73%). Table 1 depicts their baseline characteristics. Almost all patients had ECOG PS of 0 (n = 36; 97%). Several patients were affected by prostate cancer (n = 14; 38%).

**Table 1:** Baseline characteristics of patients

Characteristics	Total	
	n	%
Patients in population	37	100
Gender		
Male	27	73.0
Female	10	27.0
Age (years)		
Patients with data	37	
Mean	62.4	
SD	11.0	
Median	61.0	
Range	36–83	
Racial origin		
White	35	94.6
Black	2	5.4
Baseline ECOG		
0	36	97.3
1	1	2.7
Primary malignancies		
Bladder carcinoma	1	2.7
Breast cancer	3	8.1
Gastroesophageal junction	1	2.7
Malignant melanoma	3	8.1
Melanoma	5	13.5
Non-small-cell lung cancer	8	21.6
Pancreatic carcinoma	1	2.7
Prostate cancer	14	37.8
Renal cell carcinoma	1	2.7

Subjects with missing baseline information are excluded from the corresponding analysis

*SD* standard deviation

### Safety

Overall, 35 patients (94.6%) experienced an AE; the most common was injection site pain (62.2%) (Table 2). Other AEs experienced by at least 10% of patients included injection site erythema (45.9%), injection site swelling (43.2%), fatigue (29.7%), and nausea (18.9%).

**Table 2:** Adverse events

Patients in population	Two doses of V935 (low dose)	Three doses of V934 and two doses of V935 (low dose)	Two doses of V935 (high dose)	Three doses of V934 and two doses of V935 (high dose)	Five doses of V934 and two doses of V935 (high dose)	Total
	n (%)	n (%)	n (%)	n (%)	n (%)	n (%)
With follow-up	3 (100.0)	3 (100.0)	10 (100.0)	11 (100.0)	10 (100.0)	37 (100.0)
With one or more adverse events	3 (100.0)	3 (100.0)	8 (80.0)	11 (100.0)	10 (100.0)	35 (94.6)
Injection-site	1 (33.3)	2 (66.7)	3 (30.0)	10 (90.9)	10 (100.0)	26 (70.3)
Non-injection-site	3 (100.0)	2 (66.7)	8 (80.0)	10 (90.9)	10 (100.0)	33 (89.2)
With no adverse event	0 (0.0)	0 (0.0)	2 (20.0)	0 (0.0)	0 (0.0)	2 (5.4)
With vaccine-related^a^ adverse events	2 (66.7)	3 (100.0)	4 (40.0)	11 (100.0)	10 (100.0)	30 (81.1)
Injection site	1 (33.3)	2 (66.7)	3 (30.0)	10 (90.9)	10 (100.0)	26 (70.3)
Non-injection site	1 (33.3)	1 (33.3)	1 (10.0)	5 (45.5)	5 (50.0)	13 (35.1)
With serious adverse events	0 (0.0)	0 (0.0)	0 (0.0)	2 (18.2)	1 (10.0)	3 (8.1)
With serious vaccine-related adverse events	0 (0.0)	0 (0.0)	0 (0.0)	0 (0.0)	0 (0.0)	0 (0.0)
Who died	0 (0.0)	0 (0.0)	0 (0.0)	0 (0.0)	0 (0.0)	0 (0.0)
Discontinued^b^ due to an adverse event	0 (0.0)	0 (0.0)	0 (0.0)	0 (0.0)	0 (0.0)	0 (0.0)
Discontinued due to a vaccine-related adverse event	0 (0.0)	0 (0.0)	0 (0.0)	0 (0.0)	0 (0.0)	0 (0.0)
Discontinued due to a serious adverse event	0 (0.0)	0 (0.0)	0 (0.0)	0 (0.0)	0 (0.0)	0 (0.0)
Discontinued due to a serious vaccine-related adverse event	0 (0.0)	0 (0.0)	0 (0.0)	0 (0.0)	0 (0.0)	0 (0.0)

^a^Determined by the investigator to be related to the vaccine

^b^Study medication withdrawn

A total of 81.1% of patients experienced one or more vaccine-related AEs. Vaccine-related AEs experienced by at least 10% of patients are reported in Table 3. There were no AEs that led to discontinuation of the study. There were no vaccine-related serious AEs or vaccine-related grade 3–5 AEs. There were no DLTs in this study and no deaths were reported.

**Table 3:** Frequency of adverse events (only for incidence ≥ 10% in one or more vaccination groups)

Patients in population	Total (n = 37)
	n (%)
With one or more adverse events	35 (94.6)
With no adverse events	2 (5.4)
Injection site pain	23 (62.2)
Injection site erythema	17 (45.9)
Injection site swelling	16 (43.2)
Fatigue	11 (29.7)
Nausea	7 (18.9)
Diarrhoea	5 (13.5)
Dyspnoea	5 (13.5)
Nasopharyngitis	5 (13.5)
Arthralgia	4 (10.8)
Back pain	4 (10.8)
Cough	4 (10.8)
Dizziness	4 (10.8)
Pyrexia	4 (10.8)

### Immunogenicity

**Fig. 2:**
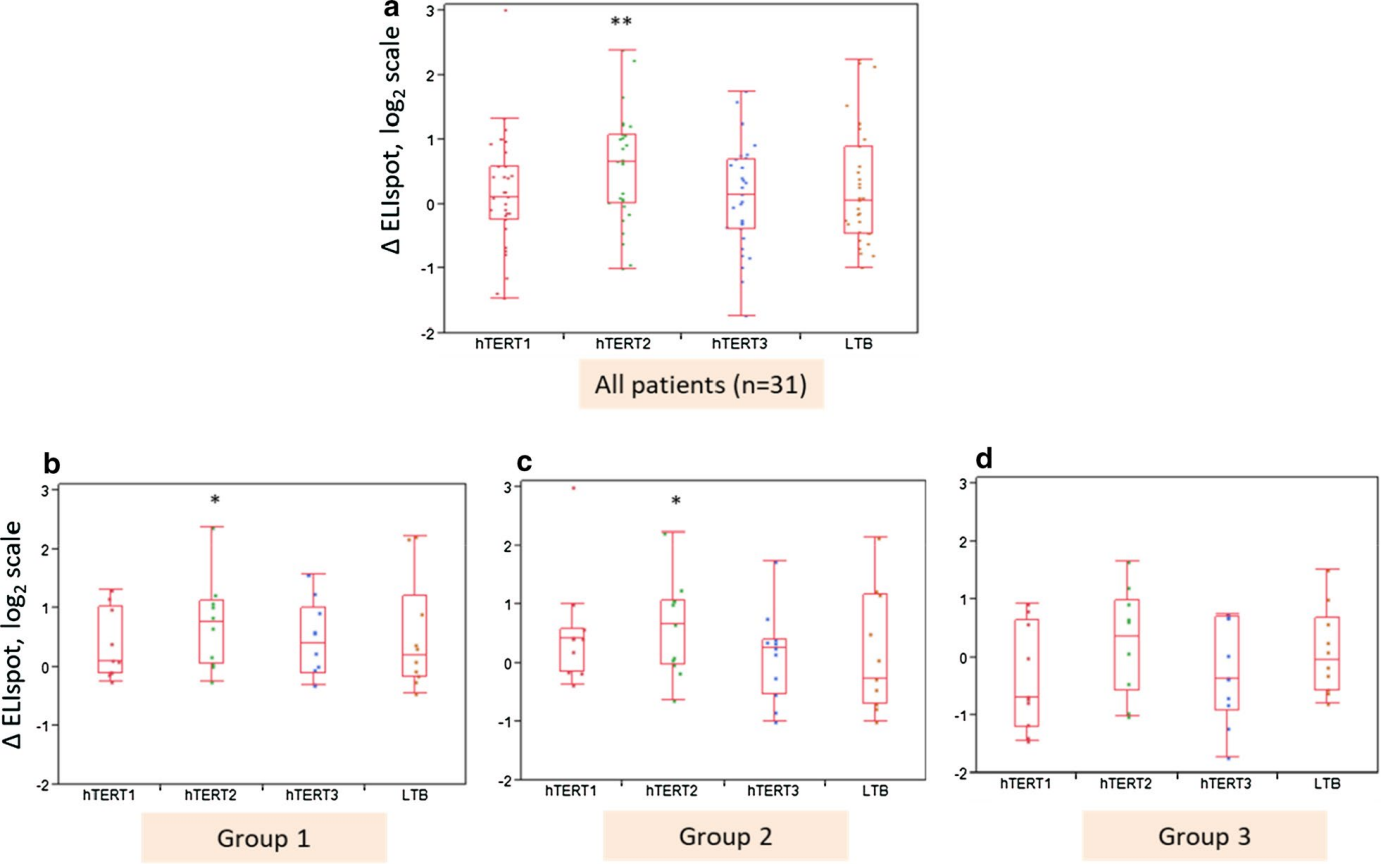
Cell-mediated immune response to hTERT peptide pools 1/2/3 and LTB peptide pool. **a**–**d** show the changes between antigen-specific ELISPOT responses post-immunization versus the baseline ELISPOT responses at pre-immunization screening (denoted by ∆ELISPOT). Box plots hTERT1/2/3 indicate hTERT peptide pools 1/2/3, respectively. Box plot LTB indicates the LTB peptide pool. Each box plot represents either all patients (**a** box plots) or individual groups 1–3 (**b**–**d**). A statistically significant increase in ELISPOT responses following hTERT immunization within each peptide pool (i.e., each boxplot) at significance levels p < 0.05 (or p < 0.01) is indicated by * or **, respectively

immunogenicity data were available for 31 patients (group 1: 10 patients, group 2: 11 patients, and group 3: 10 patients). Overall, a statistically significant increase in ELISPOT responses against peptide pool 2 was observed (p < 0.01), while no evidence of boosting was observed for peptide pools 1 and 3. This pattern was also evident when analyzing separately groups #1 and #2, but not in group #3 (Fig. 2).

**Fig. 3:**
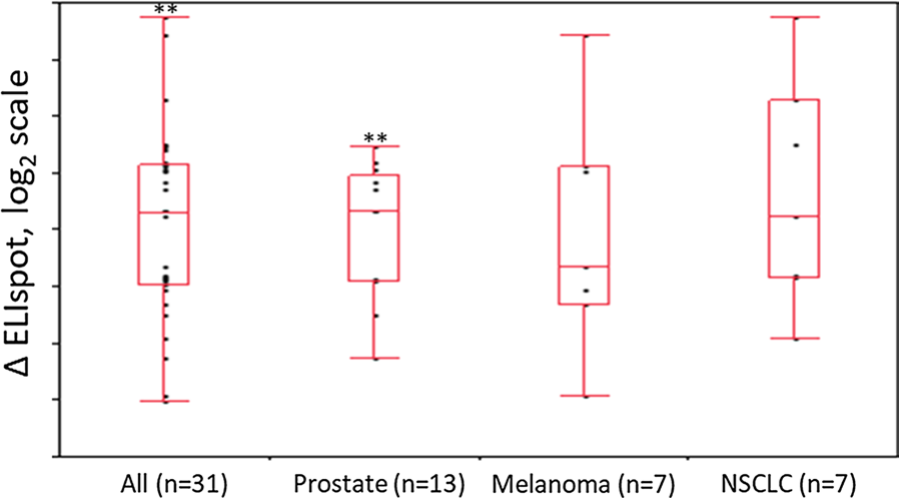
Cell-mediated immune response to hTERT peptide pool 2 for the most frequent tumor types. Cell-mediated immunity response to hTERT pool 2 increased significantly (p < 0.01) in prostate cancer patients. No statistically significant response was observed in other tumor types (here malignant melanoma patients are pooled together with melanoma patients)

The rate of response was not influenced by previous oncological therapy (seven patients among the 14 responders) or use of immunosuppressing agents (five patients among the 14 responders). There were 21 patients with no Ad6 titers at screening (< 18 units in Ad6 assay). Six patients had Ad6 titers > 18, while no data were available for four other patients. These 21 patients with no Ad6 titers at screening showed significant increase in ELISPOT responses against peptide pool 2 (p < 0.01). In patients with prostate cancer, there was a significant increase in ELISPOT response against hTERT peptide pool 2 following immunization (p < 0.01) (Fig. 3); this increased response was not influenced by previous therapy or use of immunosuppressing agents (data not shown).

## Discussion

Although being considered a promising anti-tumor strategy, to date, cancer vaccination has not shown to be a clinically useful approach in patients with severe disease [24]. This overall negative result is at least in part due to the poor effectiveness of gene transfer with currently employed strategies and, particularly, to the frequent onset of a neutralizing antibody response against viral vectors which limits the use of repeated vaccinations [11, 24]. Therefore, new immunization regimens able to reduce neutralizing antibody response and to maintain active immune response over time are needed to assess the actual potential of cancer vaccination in clinical practice.

In this pilot, phase I study, we tested the feasibility of a prime-boost immunization regimen consisting of two doses of V935 administered alone or in combination with three to five doses of V934 by EP. We believe that this approach has a strong pharmaceutical and immunological rationale. Indeed, Adenoviral vectors are highly efficacious and immunogenic against many antigens in humans (e.g., HCV, Ebola) and DNA electroporation is known to enhance gene expression and boost immune responses compared with DNA vaccination alone, since it allows a better transduction of muscle fibers and relative gene expression [11, 13].

Moreover, immunization regimens combining sequential vaccination with two different delivery systems are known to induce a more intense and durable immune response [12]. The rationale for the clinical regimen of three or five DNA doses followed by two adenovirus doses is supported by immunological proof of concept studies in transgenic mice, in rhesus monkeys and in dogs, for many antigens, including TERT [25, 26].

Due to the pilot nature of the study, we enrolled a limited number of patients, with heterogeneous characteristics and affected from different solid tumors. Overall, the safety profile of the different regimens tested in our study was good, with no severe AEs, DLTs or treatment discontinuations, and comparable across groups. As expected, the most common AEs reported were local reactions, such as pain, erythema and swelling, which are usually manageable in clinical practice.

On the other hand, immunogenicity data suggest an increased or de novo induction of hTERT-targeting response against peptide pool 2, both when all patients were analyzed and in those assigned to group #1 and #2. This finding was consistent regardless of previous therapy, use of immunosuppressing agents, or Ad6 titers at screening. Unfortunately, we have not characterized the MHC-haplotype of these patients. Pool 2 indeed contains many hTERT MHC class I predicted epitopes which were shown immunogenic both in vitro and in vivo [27] and this may be the reason why most of them responded to this region of the antigen. Additionally, the immunological response against hTERT was observed in the largest subgroup of patients, i.e. those with prostate cancer.

More recently, a genetic vaccine based on the same genetic platform (Ad6/DNA-EP) and encoding dog telomerase reverse transcriptase (dTERT) in the same configuration of V934/V935 was shown to induce strong immune response in dogs affected by B-cell lymphoma in combination with standard therapy. Most importantly, chemotherapy did not interfere with the effects of the immunotherapy and the survival of canine lymphoma was significantly augmented in comparison to chemotherapy treated subjects in three different studies [26, 28, 29] and TERT expression was a prognostic marker associated with longer survival. Interestingly, in the canine studies we adopted the Ad prime/DNA-EP boost with the rationale of inducing a strong response with Ad vectors and maintain it over time with DNA. The choice of prostate cancer patients and this inverted heterologous prime/boost regimen may represent the basis of a possible future clinical trial with V934/V935.

## Conclusion

The results of this pilot study suggest the safety and feasibility of V934/V935 hTERT vaccination in cancer patients with solid tumors. Larger studies are needed for a proper evaluation of the efficacy of this strategy and its potential impact to clinical practice.

## Data Availability

The datasets used and/or analyzed during the current study are available from the corresponding author on reasonable request.

## Abbreviations

EP: electroporation
hTERT: human telomerase reverse transcriptase
Ad6: adenoviral type 6 vector
LTB: labile enterotoxin
CMV: human cytomegalovirus
bGH: bovine growth hormone
GCP: good clinical practice
IM: intramuscularly
AE: adverse event
CTCAE: Common Terminology for Adverse Events
DLT: dose-limiting toxicity
CPK: creatine phosphor kinase
ATRC: Adult Treatment Report Cards
RECIST: Response Evaluation Criteria In Solid Tumors
dTERT: dog telomerase reverse transcriptase
ELISPOT: enzyme-linked immunospot
